# Application of Laser Scanning Confocal Microscopy for the Visualization of *M. tuberculosis* in Lung Tissue Samples with Weak Ziehl–Neelsen Staining

**DOI:** 10.3390/jcm8081185

**Published:** 2019-08-07

**Authors:** Maria V. Erokhina, Larisa N. Lepekha, Elena E. Voronezhskaya, Leonid P. Nezlin, Vadim G. Avdienko, Atadzhan E. Ergeshov

**Affiliations:** 1 Department of Pathology, Cell Biology and Biochemistry, Central Tuberculosis Research Institute, 2 Yauzskaya Alleya, 107564 Moscow, Russia; 2 Faculty of Biology, Lomonosov Moscow State University, 1–12 Leninskie Gory, 119991 Moscow, Russia; 3 Department of Comparative and Developmental Physiology, Koltsov Institute of Developmental Biology, Russian Academy of Sciences, 119334 Moscow, Russia; 4 Department of Immunology, Central Tuberculosis Research Institute, 2 Yauzskaya Alleya, 107564 Moscow, Russia; 5 Director of the Institute, Central Tuberculosis Research Institute, 2 Yauzskaya Alleya, 107564 Moscow, Russia

**Keywords:** pulmonary tuberculosis, sensitivity of *M. tuberculosis* detection in tissue, Ziehl–Neelsen staining, confocal laser scanning microscopy, polyclonal antibody, immunohistochemistry

## Abstract

One of the key requirements for the diagnosis of pulmonary tuberculosis is the identification of *M. tuberculosis* in tissue. In this paper, we present the advantages of specific fluorescent antibody labelling, combined with laser scanning confocal microscopy (LSCM), for the detection of *M. tuberculosis* in histological specimens of lung tissues. We demonstrate that the application of LSCM allows: (i) The automatic acquisition of images of the whole slice and, hence, the determination of regions for subsequent analysis; (ii) the acquisition of images of thick (20–40 μm) slices at high resolution; (iii) single bacteria identification; and (iv) 3D reconstruction, in order to obtain additional information about the distribution, size, and morphology of solitary *M. tuberculosis*; as well as their aggregates and colonies, in various regions of tuberculosis inflammation. LSCM allows for the discrimination of the non-specific fluorescence of bacteria-like particles and their aggregates presented in histological lung samples, from the specific fluorescence of labelled *M. tuberculosis*, using spectrum emission analysis. The applied method was effective in the identification of *M. tuberculosis* in lung histological samples with weak Ziehl–Neelsen staining. Altogether, combining immunofluorescent labelling with the application of LSCM visualization significantly increases the effectiveness of *M. tuberculosis* detection.

## 1. Introduction

Tuberculosis (TB) has remained a major health problem: TB is still being reported as one of the top ten causes of death worldwide [1]. The pathomorphological landscape of TB-triggered lung inflammation may be difficult to identify because of the emergence of complications from drug-resistant TB and co-morbidities such as HIV/AIDS, fungal and parasitic infections, and alveolitis. Differential diagnosis of TB is complicated because cases where the unknown etiology of diseases (such as sarcoidosis, mycobacteriosis, and granulomatous lung diseases), exhibiting a similar histological pattern, have become more common [2,3,4]. One of the key requirements for TB diagnosis is the identification of *Mycobacterium tuberculosis* (*M. tuberculosis*) in sputum and tissue, and it remains one of the most important tasks in phthisiology.

In low-income and middle-income countries, direct sputum smear microscopy (stained by Ziehl–Neelsen method) is the primary method for diagnosing pulmonary tuberculosis. The method is fast and inexpensive, but its main limitations are low sensitivity (40–60%), especially in individuals co-infected with HIV [5,6]. Another drawback of sputum analysis is that the detectable number of mycobacterium tuberculosis does not indicate the activity of the inflammatory process in the lungs [7]. In 2002, the WHO declared that it is clear that simply and sensitive diagnostic tests for TB diagnosis are needed [8].

The clinical identification of *M. tuberculosis* comprises a set of techniques (bacteriological methods, Ziehl–Neelsen staining, PCR-analysis), and the search for new methods is ongoing [9,10]. One such method is the microscopic identification of *M. tuberculosis* in histological samples. At present, two main (microscopic identification) techniques are used: (i) Ziehl–Neelsen staining of acid-resistant bacteria (the conventional method) and (ii) immunohistochemical imaging using mono- or polyclonal antibodies, directed against the components of the cell wall of *M. tuberculosis* (the newly introduced method). The Ziehl–Neelsen technique is widely used in diagnostics but has several significant limitations. First, it is not strictly specific for the *Mycobacterium tuberculosis* complex [11,12]. Second, it requires at least 10^4^–10^6^ bacteria, per slide, combined in rather large aggregates, to be reliable [13]. Additionally, the size of solitary mycobacterium is at the limit of microscopic optical resolution, and one can easily overlook acid resistant mycobacteria if their density is low and their distribution is non-homogeneous. Thirdly, the Ziehl–Neelsen staining is often negative, whereas bacteriological or PCR analysis is positive [14,15,16,17]. Fourth, the efficiency of the staining depends on the clinical stage of the disease, activity of inflammation, any previous drug therapy, and the appearance of cell wall deficient forms of bacteria, or L-forms, which are characterized by a complete or partial loss of cell wall components and by a change in cellular shape [14,18,19].

Among the methods that comprise microscopic examination, immunohistochemical (IHC) labelling is one of the most specific and sensitive for revealing interstitial *M. tuberculosis* in pulmonary and extrapulmonary TBpatients [15,16,20,21,22,23]. Histochemical reactions, that use an alkaline phosphatase or diaminobenzidine reaction, streptavidin-biotin or avidin-biotin-peroxidase complex, are mostly used for bacteria visualization [3,15,20,21,23,24,25,26]. 

Earlier, the following algorithm of analysis was developed for the search and identification of *M. tuberculosis* with the use of LSCM in lung tissue samples: (i) Automatic step-by-step scanning of a large slice (up to 10 mm^2^) and subsequent stitching of the images, selection of the regions of caseous necrosis and the perifocal zone, based on morphological heterogeneity; (ii) Automatic scanning of full volumes (20–40 μm), in selected zones, at high resolution (using the objective 63×/1.40), subsequent search for *M. tuberculosis* in the stacks, evaluation of their distribution in the tissue, selection of the zones with their different distribution, and selection of the zones for further analysis; (iii) Morphological analysis of the colonies and solitary bacteria, 3D reconstructions if necessary (see below); and (iv) If necessary, confirmation of the specificity of immunochemical labelling [27].

Fluorescent labels are widely acknowledged to be more convenient for the visualization of small subjects in microscopic preparations. However, they have not been routinely applied for *M. tuberculosis* histochemical visualization within TB patient tissues. Until now, the LSCM method has been used for the visualization of *M. tuberculosis* in experimental studies and bacterial isolates only [28,29,30].

The goal of the present work is an application of the described LSCM using the algorithm for the visualization of *M. tuberculosis* in specimens of lung tissue with weak Ziehl–Neelsen staining. We also aim to demonstrate the capabilities of fluorescent labelling of specific antibodies for the medical investigations using histological sections.

## 2. Materials and Methods

### 2.1. Preparations 

Samples of lung tissue were obtained in the Central Tuberculosis Research Institute (CTRI, Moscow, Russia) as a result of planned surgery. Informed consent was ensured from TB patients prior to surgery. All subsequent surgery material analysis was approved by the responsible CTRI Ethics Committee. The removed lung tissue was used for further histological investigation after informed consent and the patients were ensured of confidentiality. Identifying private information was omitted from the manuscript thus the patients cannot be identified from any material in the manuscript. Lung tissues samples were obtained from twenty-two TB patients with tuberculomas whose diagnosis had been confirmed by bacteriological and PCR methods. The patient age was 22–36 years (9 males and 13 females). The samples were fixed in 4% paraformaldehyde (Sigma-Aldrich, St.Louis, MO, USA) in 10 mm phosphate buffered saline (PBS, pH 7.4) overnight at 10 °C, washed 3 × 30 min in PBS, cryoprotected in 30% sucrose in PBS for 2 h, embedded in Jung tissue-freezing medium, and cut into 20–40 μm thick slices using a CM1900 cryostat (Leica, Mannheim, Germany).

### 2.2. Antibody Production

For polyclonal antibody production, rabbits were immunized with whole cells of irradiated *M. tuberculosis* H37Rv in incomplete Freund adjuvant. Sera were affinity purified by chromatography on Separose CL6B sorbent with covalently attached antigens of sonicated *M. tuberculosis* H37Rv. Cross-reactions of affinity isolated immunoglobulins were suppressed by adsorption on a similar sorbent with antigens of *M. fortuitum*. Immunoglobulins were concentrated by ultrafiltration and stored at −20 °C.

### 2.3. Antigen Retrieval

Several solutions for antigen retrieval were tested: Dako retrieval solution (Dako North America, Via Real Carpinteria, CA, USA), 5% Triton, 50% methanol and 70% methanol in PBS. The specimens were preincubated in 5% Triton in PBS during 6 h at 10°C, washed in PBS, and incubated in an antigen-retrieval solution for 1 h at room temperature. The best results were obtained after combination of preincubation in 5% Triton with subsequent treatment with methanol. Free-floating slices therefore were transferred through ascending-descending methanol series (30–50–70–50–30% in PBS, 10 min in each solution, at room temperature) and washed in PBS (3 × 10 min).

### 2.4. Immunostaining 

Primary antibodies (AB) were diluted 1:200–1:500 (from the initial concentration of 1.14 mg/mL) with PBS supplemented with 1% Triton X-100 (TX) and 0.25% BSA. Preincubation in 5% Triton allowed use 1:500 primary antibody dilution without loss of sensitivity. Slices were incubated overnight at 10 °C, washed 3 × 10 min in PBS-TX and then incubated with goat-anti-rabbit-Alexa-488 or goat-anti-rabbit-Alexa-555 IgG solutions (Molecular Probes, Eugene, OR; A-11008, A-21428, diluted 1:800). Finally, the slices were washed 3 × 10 min in PBS, immersed in 50% glycerol for 20 min and mounted on microscope slides in 80% glycerol.

### 2.5. Controls

**Figure 1:**
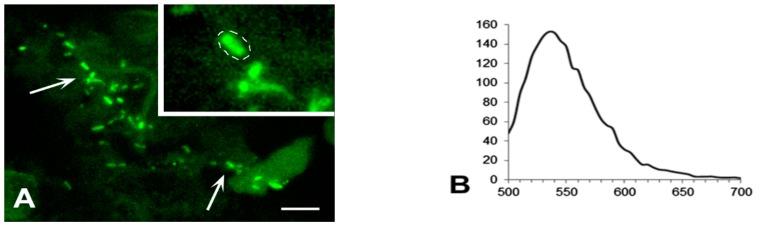
Immunolabelling of *M. tuberculosis* in the lung tissue of mice, infected with the strain H37Rv. (**A**) The colony of *M. tuberculosis* (arrows). Inset: High magnification of the bacteria, selected for measuring of an emission spectrum (dotted line). Scale bar = 5 μm. (**B**) The emission spectrum of the selected bacteria is similar to the spectrum of Alexa-488. The abscissa shows emission wavelength, nm; the ordinate shows relative brightness.

*M. tuberculosis* strain H37Rv was originally obtained from the Institute Pasteur, Paris, France. Male BALB/c inbred mice (22–23 g of body weight) were used. To test the specificity of the antibody, we used a classical mice model of generalized tuberculosis. Mice were bred under conventional conditions at the Animal Facilities of the CTRI, in accordance with the guidelines from the Russian Ministry of Health #755, NIH Office of Laboratory Animal Welfare (OLAW) Assurance #A5502. Water and food were provided ad libitum. All experimental procedures were approved by the CTRI animal care committee (IACUC protocols #4). Mice were infected by the respiratory route with 100 viable CFU/lung using an inhalation exposure system (Glas-Col, Terre Haute, IN). All animals were divided into 2 groups: Control (*n* = 5) and infected animals (*n* = 5). The animals were removed from the experiment after 21 days after infection. Slices of the lungs were labeled as described above and analyzed using confocal microscopy. Aggregates of rod-shaped mycobacteria were detected in the lung tissue (Figure 1A). As expected, the spectrum of fluorescence confirmed labeling with Alexa-488, which is conjugated to the secondary antibody employed for visualization of the primary anti- *M. tuberculosis* antibody (Figure 1B). Replacement of the secondary antibody with the one conjugated with Alexa-555 gave similar results.

Negative controls included either the omission of the primary antiserum from the staining protocol or its replacement with normal rabbit serum. Both resulted in complete loss of specific staining. Fluorescence spectra of inclusions that were present in the tissue confirmed the lack of specific labeling in the preparations processed without the primary antibody. No positive signal was detected in the preparations of control animals.

### 2.6. Ziehl–Neelsen Staining

For Ziehl–Neelsen staining, 5 µm thick sections were cut from the tissue paraffin-embedded tissue block and was performed according to the standard protocol [13]. In brief, tissue sections were deparaffinized by washing in decreasing ethanol concentrations (from 96% to 70%) and heat fixed. Then the specimens were stained with carbol-fuchsin for 4 min, washed, and incubated with HCl until the stain completely dissolved. Counterstaining was performed with Brilliant Green for 20 s. The sections were air dried after thorough washing. The number of solitary bacteria and bacteria colonies were detected visually on each preparation and counted on 500 µm^2^. Tissue samples in which the Ziehl–Neelsen demonstrated weak staining (it revealed only single bacteria and rare colonies) were selected for further immunohistochemical staining (Supplementary Table S1).

### 2.7. Microscopy and Image Processing

Images of Ziehl–Neelsen staining were captured using Leica DM1000 LED microscope (Leica, Mannheim, Germany) with N PLAN 100×/1.25 Oil objective and fitted with DFC 295 video camera (Leica, Mannheim, Germany). The immunohistochemical preparations were examined using laser scanning confocal microscopes TCS SPE, TCS SP5 (Leica, Mannheim, Germany), and FV10i (Olympus, Tokyo, Japan) equipped with appropriate excitation lasers and barrier filters. Serial optical sections were acquired and saved as 3D stacks and maximal projection. 

The number of individual bacteria was counted automatically on selected 100 µm^2^ on each selected 20 µm-thickslice (maximal optical projection, n = 12) using ImageJ software (NIH, Bethesda, MD, USA). Each of 20 µm-thick slices was divided to four 5 µm-thinoptical sections where the number of visible bacteria (on each of the 5 µm sections) was automatically calculated (using ImageJ) and normalized to the total number of bacteria counted on the 20 µm slice. Statistical analysis of bacteria manifestation variability and graphs were made using Microsoft Excel 2010. 

Deconvolution and three-dimensional, rotatable reconstructions were generated from an image stack and analyzed using the Leica LAS AF (Leica Microsystems, Mannheim, Germany) and ImageJ (NIH, Bethesda, MD, USA) software. For illustrations, a series of optical sections were projected into one image with greater focal depth and imported into PhotoShop CC (Adobe, San Jose, CA, USA). Only brightness and contrast were adjusted as needed. Fluorescence spectra were compared with the standard spectra of Alexa-488 and Alexa-555.

### 2.8. Statistical Analysis

Statistical processing was carried out using STATISTICA 10 software (TIBCO Software Inc., Palo Alto, CA, USA). To compare independent samples, the Mann–Whitney test was used. The differences in the control and experimental samples were considered significant for *p* < 0.01.

## 3. Results 

### 3.1. Detection of M. tuberculosis in Histological Sections Using Ziehl–Neelsen Staining

**Figure 2:**
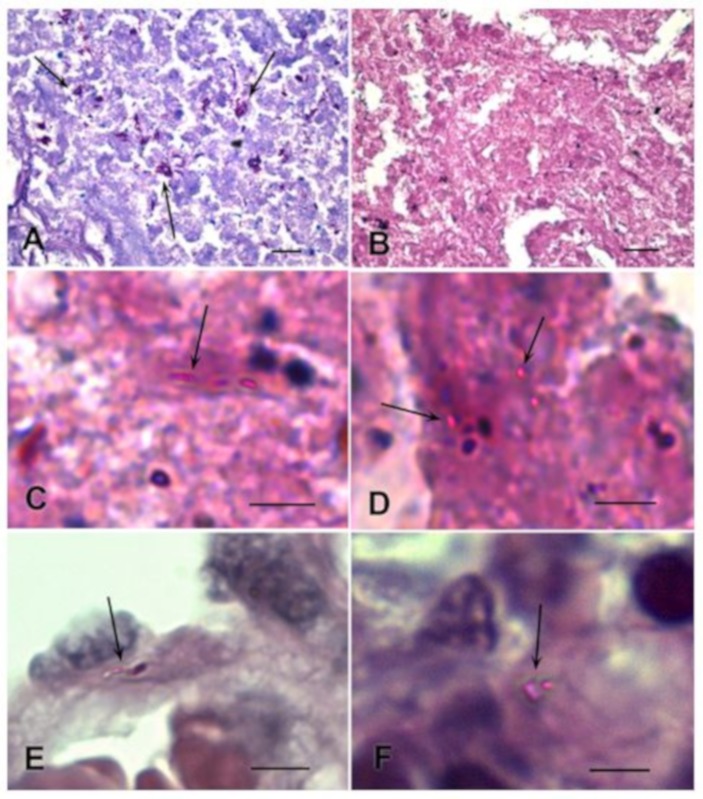
Ziehl–Neelsen staining of *M. tuberculosis*, in the lung tissue of TB patients, with different bacteria content. (**A**) Aggregations of *M. tuberculosis* in a region of caseous necrosis, in a sample of lung tissue with high bacterial content. (**B**–**F**) Examples of lung tissue with low bacterial content, selected for immunolabelling. (**B**) At low magnification, aggregations of *M. tuberculosis* are not visible. (**C**,**D**) High magnification of single small colonies of rod-shaped and rounded bacteria (arrows) in a region of caseous necrosis. (**E**,**F**) High magnification of single rod-shaped and rounded bacteria in the perifocal region. Scale bars: (**A**,**B**) = 10 µm; (**C**–**F)** = 5 µm.

The Ziehl–Neelsen method is a traditional morphological method, used to visualize acid-resistant bacteria, using light microscopy. An overview of regions of caseous necrosis demonstrates a difference in the Ziehl–Neelsen staining of *M. tuberculosis*, within lung tissue with high (Figure 2A), and low (Figure 2B) bacterial content. 

Figure 2A shows Ziehl–Neelsen staining of a preparation with large aggregates, which allows one to confirm a diagnosis of TB, if additional morphological and clinical assessments are present. In contrast, Ziehl–Neelsen staining did not allow an unambiguous identification of *M. tuberculosis* and their colonies in samples of lung tissue with a low bacterial content (Figure 2B). High magnification is necessary to recognize rod-shaped bacteria in the region of caseous necrosis (Figure 2C) and in the perifocal region (Figure 2E) of these samples. Solitary rounded bacteria, and small groups of 2–7 bacteria, could also be seen at high magnification only (Figure 2D,F). The search for the stained mycobacteria in these preparations was often a difficult and lengthy process because of the small number of *M. tuberculosis*. In general, Ziehl–Neelsen staining did not allow the unambiguous diagnosis of “tuberculosis” according to this criterion. We selected the samples with low *M. tuberculosis* content, as revealed by Ziehl–Neelsen staining (median = 7.5; 25–75% = 1.5–16), for further immunofluorescence labelling (Supplementary Table S1).

### 3.2. Detection of M. tuberculosis Using Immunofluorescence Labelling and LSCM

The region of caseous necrosis and the perifocal zone were determined in an overview image that resulted from the stitching of intermediate images, and the zones for further analysis were selected according to the described algorithm [23].

#### 3.2.1. Analysis of *M. tuberculosis* in the Caseous Necrosis Region

**Figure 3:**
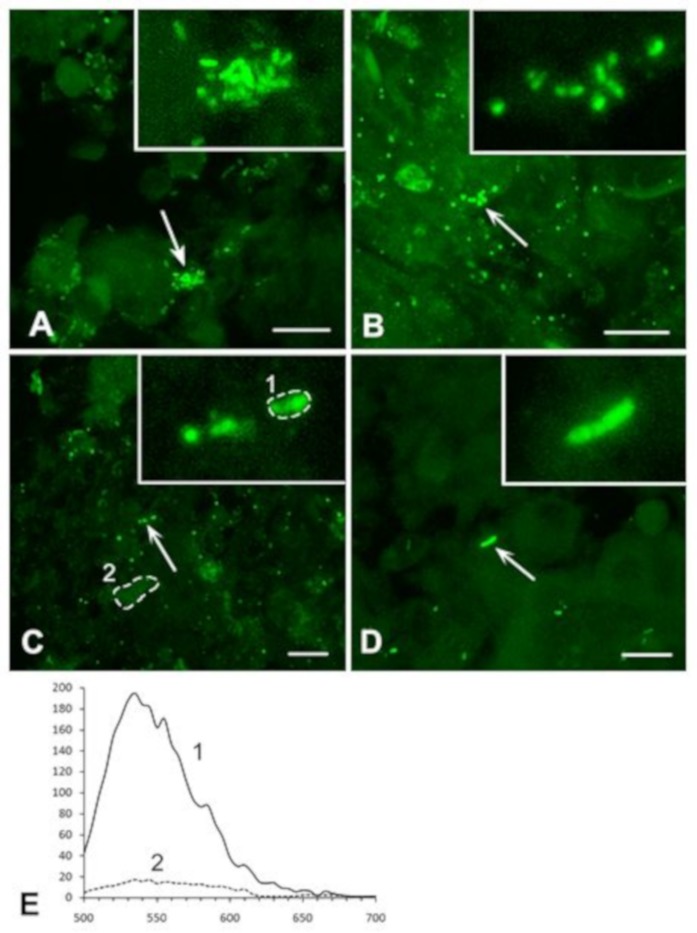
Immunolabelling of *M. tuberculosis* in lung tissue; the region of caseous necrosis. Small aggregations (**A**,**B**), colonies (**C**), and solitary bacteria (**D**) were detected. Insets: High magnification of the structures indicated by arrows. Scale bars = 10 µm. (**E**) The emission spectrum of the fluorescent structure, defined by the dotted line, in (**C**): (**1**) Corresponds to the spectrum of the fluorescent marker Alexa-488, while the spectrum of surrounding tissue (**2**) is different. The abscissa shows emission wavelength, nm; the ordinate shows relative brightness.

In the region of caseous necrosis, the density of bacteria varied, from multiple groups to solitary bacteria (Figure 3).

At high magnification, *M. tuberculosis* were detected as aggregates of rod-shaped bacteria and rounded forms (Figure 3A,B), well-defined chain-like colonies of 3–7 bacteria (Figure 3C,D), and solitary rod-like 1 to 2 µm-longbacteria (Figure 3D). Aggregates or colonies of 0.5 μm rounded fragments (or forms) of *M. tuberculosis* and rod-like forms were most common. Due to the small size of the bacteria, the solitary fluorescent structures required additional spectral analysis, to separate the antibody-labelled bacteria from the non-specific fluorescent particles. An emission spectrum of a rod-like structure, shown in Figure 3C, was equal to the spectrum of Alexa-488, the fluorescent marker of the secondary antibody, whereas an emission spectrum of the surrounding tissue was different (Figure 3E).

Immunohistochemical labelling revealed *M. tuberculosis* colonies in all of the selectedsamples of lung tissue with weak Ziehl–Neelsen staining (Supplementary Table S1). 

#### 3.2.2. Analysis of *M. tuberculosis* in the Perifocal Region

**Figure 4:**
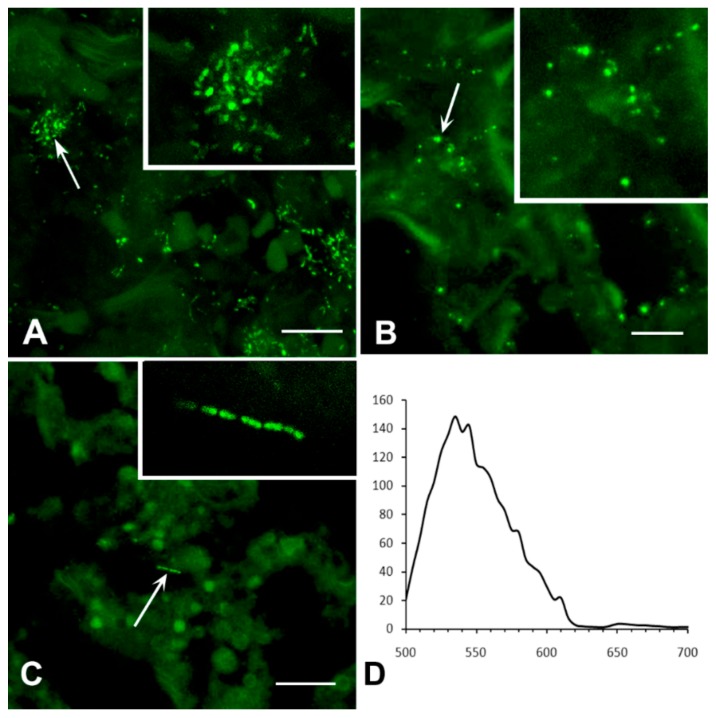
Immunolabelling of *M. tuberculosis* in lung tissue; the perifocal region of TB inflammation. *M. tuberculosis* are presented as large (**A**) or small aggregations (**B**), and solitary chain-like colonies (**C**) Insets: High magnification of the structures indicated by arrows. Scale bars = 10 µm. (**D**) The emission spectrum of the chain-like colony, shown in (**C**), corresponds to the spectrum of the fluorescent marker Alexa-488. The abscissa shows emission wavelength (nm); the ordinate shows relative brightness.

Rounded and rod-shaped bacteria, of 0.5–1 μm in length, were identified in the perifocal zone. The overview images revealed that bacterial distribution varied, from a relatively high density (Figure 4A) to dispersed (Figure 4B), and even to single chain-like colonies only (Figure 4C).

It should be noted that aggregates and clusters of *M. tuberculosis*, as well as solitary and chain-like bacteria, were more frequently detected in the perifocal region than in the region of caseous necrosis. All immunohistochemically detected bacteria were rounded or rod-shaped (Figure 4A–C insets). The specificity of the immunolabelling of the solitary chain of *M. tuberculosis* was additionally confirmed by its emission spectrum similarity to that of the specific fluorescence marker, Alexa 488 (Figure 4D).

### 3.3. Exclusion of Non-Specific Fluorescence from the Analysis

**Figure 5:**
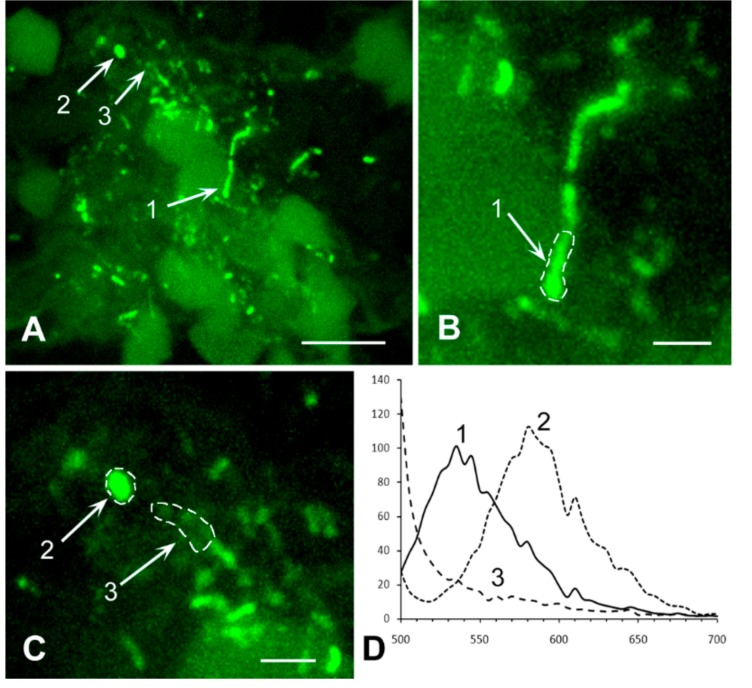
Immunolabelling of *M. tuberculosis* in lung tissue with specific staining and non-specific fluorescence. A low magnification image (**A**) shows aggregations of rounded and rod-shaped fluorescent structures, whereas specific and non-specific fluorescence is indistinguishable. High magnification of the selected structures is shown in (**B**,**C**), and their emission spectra (the ROIs are indicated by dotted lines) are presented in (**D**). Specific labelling, with the emission spectrum similar to that of Alexa-488 (**1**) can be distinguished from non-specific fluorescence, with different emission spectra (**2**,**3**). Scale bars: (**A**) = 10 µm; (**B**,**C**) = 2 µm. The abscissa shows emission wavelength (nm); the ordinate shows relative brightness.

One of the advantages of laser confocal microscope is that emission spectra can be obtained from a selected area (region of interest, ROI, Figure 5) of an image. This allows the specific fluorochrome used to label *M. tuberculosis* to be differentiated from the non-specific fluorescence of small particles which are often present in tissues. It is important that this discrimination is made, in diagnostics, for two reasons: (i) The visual similarity in the form and glow between *M. tuberculosis* and small particles; and (ii) the small size of *M. tuberculosis* (0.5–1 μm) and its various morphological forms (rod-shaped and rounded). We analyzed the emission spectra of different fluorescent aggregates in lung tissue (Figure 5A) and compared these with the emission spectrum of Alexa-488, which we used to visualize the antibody against *M. tuberculosis*. 

The emission spectra allowed an unambiguous discrimination of the specific fluorescence of the secondary antibody and non-specific fluorescence of the particles, which are similar to *M. tuberculosis* in shape and size (Figure 5B,C). Despite the similarities in morphology, the spectral analysis of the selected ROIs (Figure 5D) eliminated the structures with non-specific fluorescence, from the morphological base of the diagnosis. 

Thus, the combination of immunofluorescent labelling, high resolution scanning using a laser confocal microscope, and the acquisition of emission spectra allowed us to confirm the presence of both aggregates and single *M. tuberculosis* by their size, shape and specific emission spectrum even in lung tissue samples with weak Ziehl–Neelsen staining. Our results support that such a diagnostic approach may increase the sensitivity and reliability of the diagnosis.

### 3.4. Analysis of Bacterial Density

**Figure 6:**
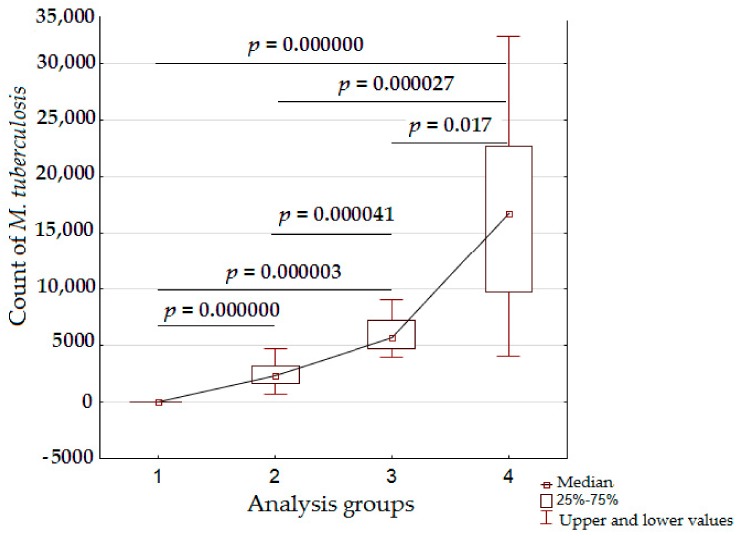
Comparison of the detected density of *M. tuberculosis* in staining according to Ziehl–Neelsen and immunohistochemical staining of samples of 5, 20, and 40 μm-thick lung tissue optical slices. Analysis groups: (**1**)Ziehl–Neelsen staining, 5 µm-thick slices; 2–4, ICC-staining; (**2**) 5 µm-thick slices; (**3**) 20 μm-thick slices; (**4**) 40 μm-thick slices. The abscissa shows number of observations per slices 500 μm^2^; the ordinate shows the group of analysis.

We compared the number of visible bacteria in an optical section of 5, 20, and 40 µm-thick after IHC staining. The analysis showed that the number of detected bacteria increased 7-fold when using optical sections to 40 μm, compared with sections to 5 μm (median = 16695; 25–75% = 9765–22680 against the median = 2300; 25–75% = 1610–3140) and 3 times, compared with sections of 20 μm thickness (median = 16,695; 25–75% = 9765–22,680 vs. median = 5717; 25–75% = 4767–7180) (Figure 6). It can be stated that the IHC method is a more effective way of detecting *M. tuberculosis* compared to Ziehl–Neelsen staining, and its effectiveness depends, among other things, on the thickness of the histological sections.

### 3.5. Detailed Analysis of Bacterial Density, Colony Forms, and Individual Bacterial Morphology

We compared a number of visible bacteria, as well as colony forms and individual bacterial morphology, in thick 20 µm slices and thin 5 µm optical sections, all produced from the same preparations.

**Figure 7:**
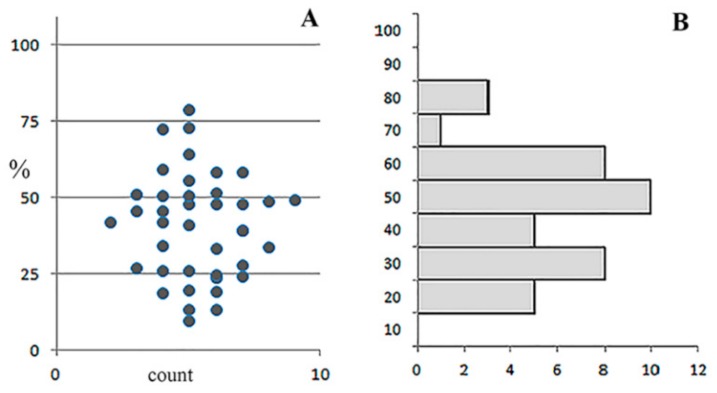
Representation of immunopositive *M. tuberculosis* on 5 µm-thick slices and 20 µm-thick optical sections, produced from the same preparations. (**A**) Number of visible bacteria on 5 µm optical sections, normalized to overall 20 µm slices. Each point represents one optical section (500 μm^2^). (**B**) Ranging histogram, representing variability of the section with high and low bacteria density. The abscissa shows number of observations; the ordinate shows a number of bacteria, % from the 20 µm sample.

Obviously, more bacteria were visible on the 20 µm slices as opposed to each 5 µm optical section (Figure 7A).

However, the bacterial distribution that was displayed in the 5 µm sections varied greatly, from 10% to 70% of the overall number displayed in the respective 20 µm slices (Figure 7B), depending on the optical section level and bacterial distribution within the lung tissue. 

**Figure 8:**
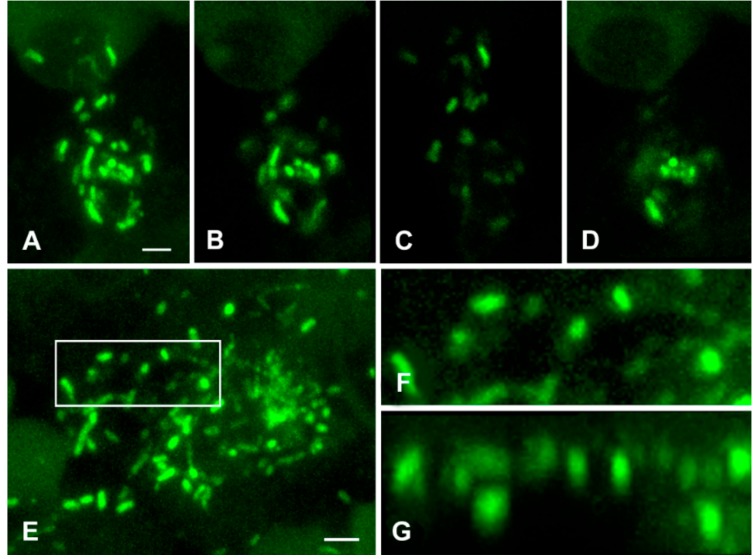
Immunolabelling of *M. tuberculosis* in the perifocal region of lung tissue. (**A**) The maximum intensity projection of the aggregate of bacteria (20 µm-thick optical sections). (**B**–**D**) 2 µm-thick optical sections through different parts of this aggregate. (**E**) The aggregate with rounded and elongated, but not rod-shaped, structures. (**F**) High magnification of rounded structures in the region defined in (**E**) (rectangle). (**G**) Side view of the same region (XZ projection after blind deconvolution) shows that the structures are really rounded. Scale bar = 2 µm.

The manifestation of *M. tuberculosis* clusters also depended on the thickness of the slice. Figure 7 shows optical sections of the representative cluster of *M. tuberculosis*, which was obtained from the 20 µm-thickpreparation. The aggregate consisted of 1 to 2 µm-long, rod-shaped bacteria. The number, shape and fluorescence intensity of *M. tuberculosis* were manifested differently in each 2 µm-thickoptical section. The maximum intensity projection showed a large aggregate of multiple bacteria (Figure 8A).

However, different parts of the same aggregate can be manifested as a group of single bacteria or their fragments depending on the optical section (Figure 8B–D). Thus, the *M. tuberculosis* density and shape of the colony in conventional histological 2 to 5 µm-thick slices depends upon which part of the colony ends up in the slice; thence, this may lead to an incorrect diagnosis. An advantage of LSCM is that it allows the investigation of thick histological slices (up to 20–40 μm). 

In addition, 3D reconstructions allow an analysis of the structure of individual bacteria and their aggregates in different projections. We used XY and XZ projections for representative colonies (Figure 8E and Supplement 1 for 3D movie). In one sample, the rod-shaped bacteria looked rounded (see Supplement 1 for 3D movie). We expected that, in another sample, the bacteria that looked round-shaped at XY projection would actually be rod-shaped at the XZ projection. However, a 3D analysis confirmed, unambiguously, that the rounded bacteria retained their form in all projections; thus, proving the presence of atypical, rounded bacterial forms within the TB lung tissue (Figure 8E–G, Supplementary movie S1).

## 4. Discussion

In this paper, we have demonstrated the application of an LSCM technique combined with fluorescent antibody labelling for the detection of *M. tuberculosis* in histological specimens of lung tissue of pulmonary TB patients. Our results showed a high efficiency of such a methodical approach for detection of *M. tuberculosis* in tissue samples with weak Ziehl–Neelsen staining. The efficiency and sensitivity of this methodical approach increases with the use of thick (up to 40 µm) histological sections.

The efficiency of the immunohistochemical method as it applies to the detection of *M. tuberculosis* in histological samples from patients with pulmonary and extrapulmonary forms of TB has been regularly demonstrated and discussed. Many authors indicated that the immunohistochemical method is more sensitive than the traditional Ziehl–Neelsen-staining [15,16,20,24,25]. Comparisons of immunohistochemical and Ziehl–Neelsen methods have shown that the latter becomes ineffective when the concentration of *M. tuberculosis* is lower than 10^4^ mycobacteria/slide, whereas immunohistochemical methods can detect lower than 10 mycobacteria per slide [20]. Studies conducted by Goel and Budhwar showed that the specificity of the immunohistochemical method is 64–100% versus 0–44% for the Ziehl–Neelsen staining for diagnosing extrapulmonary tuberculosis [16]. It should be noted that, traditionally, this immunohistochemical technique employs paraffin embedding, microtome sectioning into 3–5-μm-thick slices, histochemical labelling of the secondary antibodies, and further analysis using light microscopy [15,20,23,24,25,26,31,32]. Such a method for *M. tuberculosis* detection permits: (i) Imaging of the distribution of *M. tuberculosis* in different zones of TB inflammation; (ii) detection of the presence of extra- and intracellular *M. tuberculosis* forms; (iii) imaging of *M. tuberculosis* in different types of cells; and (iv) visualization of morphologically diverse *M. tuberculosis* forms, such as rod-shaped or globular bacteria, as well as bacterial wall fragments [29,33]. In this case, histochemical labelling of the secondary antibodies can lead to the appearance of some artefacts: (i) *M. tuberculosis*, and their aggregates, are sometimes indistinguishable from inclusions of hemosiderin and soot particles in lungs, which are often present in biopsy material; and (ii) an experienced pathologist is required to distinguish between non-specific staining and morphologically modified forms of *M. tuberculosis* and their fragments [24]. 

Currently, in the diagnosis of tuberculosis, the use of light microscopy is more common and widespread than the use of fluorescence microscopy. At the same time, the emergence of laser confocal microscopy has considerably expanded the capabilities of the analysis of histological preparations. In our work, we confirmed the advantages of the immunohistochemical method before Ziehl–Neelsen staining for the *M. tuberculosis* detection on standard histological sections of up to 5 μm thick. We also demonstrated that the undoubted advantage of LSCM is the possibility of using thick histological sections: This increases the array of *M. tuberculosis* detected by 3 times (in the case of 20 μm) and 7 times (in the case of the thickness of sections up to 40 μm). In addition, the use of spectroscopy made it possible to exclude bacteria-like structures with nonspecific autofluorescence from the analysis. Although the application of the thickest sections showed a more outstanding result, in our opinion it is more preferable to use sections with a thickness of not more than 20 μm, as this allows a more thorough analysis.

It should be noted that immunohistochemical methods for histological *M. tuberculosis* labelling have this limitation: The absence of highly specific antibodies that would not have cross-specificity to other *Mycobacteria tuberculosis* complex. Despite this, many authors stress that these limitations do not diminish the superior diagnostic value of the immunochemical method and they acknowledge its high sensitivity when mono- or polyclonal antibodies are used to detect *M. tuberculosis* [7,20,21,23,34]. 

Studies in which the analysis of specific dyes fluorescence, with the help of LSCM, is carried out in order to increase the efficiency of diagnostic approaches are already emerging. It was shown that the use of a laser confocal microscope makes it possible to detect amyloid in different tissues after staining with Congo red (CR), or Thioflavin-T (ThT). The authors used 2, 4, and 8 μm sections and confirmed the specificity of staining by spectroscopy [35]. In this study it was shown that LSCM reduces the disturbing effects of sample thickness and tissue autofluorescence on the detection of amyloid, with substantially less false positive and false negative diagnoses of amyloidosis. In particular, amyloid detection by ThT-LSCM was found to be more reliable than by ThT-epiFM, CR-epiFM (FM, fluorescencemicroscopy). The authors come to the important conclusion that ThT-LSCM is a sensitive screening method, which is easy to standardize. 

## 5. Conclusions

The methodological application of fluorescent immunohistochemistry and LSCM demonstrate the quick and reliable detection of *M. tuberculosis* in surgical samples. The use of large and thick slices enabled the selection of regions for further detailed analysis in both XY and XZ projections to detect the aggregates, colonies, and the shape of the single bacteria in the selected regions. A great advantage of the LSCM analysis is the exclusion of non-specific fluorescence and confirmation of the specificity of immunolabelling, by acquiring emission spectra of the used fluorescent markers. To summarize, the combined immunochemical labelling with subsequent LSCM 3D analysis permits a greater likelihood of the detection of low density of bacteria and their colonies, allowing us to visualize solitary *M. tuberculosis* and determine their detailed morphological characteristics. This is critical for TB diagnostics in histological samples of TB patients (especially in cases of weak Ziehl–Neelsen staining or modified forms of *M. tuberculosis*), as well as for experimental studies. We consider that the increasing availability of microscopic tools, particularly LSCMs, in medical facilities means that such an approach may increasingly be adopted as an efficient instrumental method for both medical research and clinical diagnostics.

## Supplementary Materials

The materials are available online at https://www.mdpi.com/2077-0383/8/8/1185/s1.
