# Supplementary material for: Application of Laser Scanning Confocal Microscopy for the Visualization of M. tuberculosis in Lung Tissue Samples with Weak Ziehl–Neelsen Staining
Source: J Clin Med. 2019 Aug 7;8(8):1185. doi: 10.3390/jcm8081185 (PMC6723956; doi:10.3390/jcm8081185)
Supplement: Supplementary file 1 [file jcm-08-01185-s001.zip › Table S1.docx]

**Тable S1** Representative samples of patient preparations stained using Ziehl–Neelsen method.

| **ID Sample** | **region** | **Number of bacteria,**  **500 µm^2^** | | **Selected**  **for IHC** | **IHC-positive** | **ID**  **Sample** | **region** | **Number of bacteria,**  **500 µm^2^** | | **Selected**  **for IHC** | **IHC-positive** |
| --- | --- | --- | --- | --- | --- | --- | --- | --- | --- | --- | --- |
|  |  | **colonies** | **solitary** |  |  |  |  | **colonies** | **solitary** |  |  |
| 1/0394 | CN | 0 | 0 |  |  | 12/0454 | CN | 1 | 2 | **+** | **+** |
| 1/0396 | CN | 0 | 0 |  |  | 12/0455 | CN | 0 | 5 |  |  |
| 2/0408 | PF | 0 | 12 | + | + | 13/0456 | CN | 0 | 6 | **+** | **+** |
| 2/0411 | PF | 0 | 15 |  |  | 13/0457 | CN | 1 | 14 |  |  |
| 3/0578 | PF | 0 | 3 | **+** | + | 14/0458 | CN | 2 | 10 | **+** | **+** |
| 4/0608 | PF | 10 | 102 |  |  | 14/0459 | CN | 3 | 17 |  |  |
| 4/0609 | PF | 7 | 63 |  |  | 14/0460 | CN | 4 | 18 |  |  |
| 4/0610 | PF | 19 | 117 |  |  | 15/0419 | PF | 0 | 0 |  |  |
| 5/0611 | PF | 0 | 0 |  |  | 15/0420 | PF | 0 | 0 |  |  |
| 6/0615 | PF | 9 | 85 | **+** | + | 15/0723 | CN | 0 | 0 |  |  |
| 6/0617 | PF | 4 | 50 |  |  | 16/0724 | PF | 0 | 0 |  |  |
| 7/0621 | CN | 2 | 4 | **+** | + | 16/0726 | PF | 0 | 0 |  |  |
| 7/0622 | CN | 1 | 22 |  |  | 17/0727 | CN | 0 | 0 |  |  |
| 7/0623 | CN | 1 | 9 |  |  | 17/0728 | PF | 0 | 0 |  |  |
| 8/0624 | CN | 48 | 5 |  |  | 18/0729 | CN | 0 | 0 |  |  |
| 8/0625 | CN | 40 | 195 |  |  | 18/0730 | CN | 0 | 0 |  |  |
| 8/0626 | CN | 36 | 136 |  |  | 18/0731 | CN | 0 | 0 |  |  |
| 9/0423 | CN | 0 | 0 |  |  | 19/0732 | CN | 0 | 0 |  |  |
| 9/0424 | CN | 0 | 0 |  |  | 19/0733 | CN | 0 | 0 |  |  |
| 9/0425 | PF | 0 | 0 |  |  | 20/0013 | PF | 0 | 0 |  |  |
| 10/0428 | CN | 0 | 0 |  |  | 20/0012 | PF | 0 | 2 | **+** | **+** |
| 10/0429 | CN | 0 | 0 |  |  | 20/0011 | PF | 0 | 0 |  |  |
| 10/0430 | CN | 0 | 0 |  |  | 21/0009 | PF | 0 | 1 | **+** | **+** |
| 11/0431 | CN | 0 | 0 |  |  | 21/0008 | PF | 0 | 1 |  |  |
| 11/0432 | CN | 0 | 0 |  |  | 22/0005 | PF | 1 | 0 | **+** | **+** |
| 11/0433 | CN | 0 | 0 |  |  | 22/0002 | PF | 0 | 4 |  |  |
|  | CN – caseous necrosis region; PF – perifocal region. | | | | | | | | | |  |
